# Relationships Between Glymphatic System Activity and Tau Burden, Dopaminergic Impairment, Abnormal Glucose Metabolism in Progressive Supranuclear Palsy

**DOI:** 10.1111/cns.70284

**Published:** 2025-02-18

**Authors:** Fangyang Jiao, Qingmin Wang, Jiayi Zhong, Huamei Lin, Jiaying Lu, Luyao Wang, Min Wang, Fengtao Liu, Jiehui Jiang, Chuantao Zuo

**Affiliations:** ^1^ Department of Nuclear Medicine & PET Center, National Clinical Research Center for Aging and Medicine & National Center for Neurological Disorders, Huashan Hospital Fudan University Shanghai China; ^2^ Institute of Biomedical Engineering, School of Life Science Shanghai University Shanghai China; ^3^ Department of Neurology, National Clinical Research Center for Aging and Medicine & National Center for Neurological Disorders, Huashan Hospital Fudan University Shanghai China

**Keywords:** dopamine system, glucose metabolism, glymphatic system, progressive supranuclear palsy, tau protein

## Abstract

**Background:**

Progressive supranuclear palsy (PSP) is a primary tauopathy characterized by dopaminergic impairment and abnormal glucose metabolism. The glymphatic system can promote the elimination of tau protein. The association between glymphatic function and pathological hallmark in neuroimaging remains unknown.

**Methods:**

Diffusion tensor imaging (DTI) and positron emission tomography (PET) scanning with ^18^F‐Florzolotau, ^18^F‐FPCIT, and ^18^F‐FDG were performed in PSP patients. DTI analysis along the perivascular space (ALPS) index was computed to assess glymphatic function, while the semi‐quantitative value was employed to measure tau burden and dopaminergic impairment. The PSP‐related pattern (PSPRP) served as an indicator of abnormal metabolic brain network activity.

**Results:**

PSP patients exhibited changes in ALPS index and tau deposition. ALPS index, tau deposition, and PSPRP expression showed significant correlations with clinical scores. Additionally, ALPS index was correlated with tau deposition and PSPRP expression. However, neither ALPS index nor the clinical scores were correlated with striatum dysfunction. Finally, tau deposition in subcortical regions and PSPRP expression exhibited mediating effects between ALPS index and clinical scores.

**Conclusion:**

The glymphatic dysfunction is associated with tau deposition and abnormal metabolic brain network activity and is independent of dopaminergic impairment in PSP.

## Graphical abstract

PSP patients are characterized by a remarkably decreased ALPS‐index, tau accumulation, dopaminergic dysfunction, and abnormal glucose metabolism in the brain. The glymphatic dysfunction is associated with tau deposition and abnormal metabolic brain network activity and is independent of dopaminergic impairment in PSP.

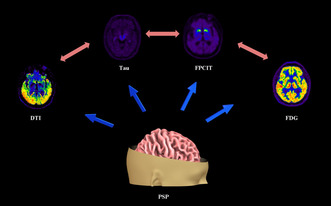

## 1 Introduction

Progressive supranuclear palsy (PSP) is a rare neurodegenerative disorder characterized by the 4‐repeat form of tau (4R tau) tauopathy [1]. The most common manifestations of PSP include vertical supranuclear ophthalmoplegia, axial dystonia, and postural instability [2]. So far, the gold standard for diagnosis is based on the neuropathological examination, which reveals the presence of 4R tau in subcortical nuclei in PSP. Previous studies have demonstrated a direct link between clinical severity and the underlying tau pathology in PSP [3, 4]. The glymphatic system is a novel mechanism that facilitates the elimination of tau protein and maintains dynamic balance in the brain. The system operates by allowing cerebrospinal fluid to flow into the brain parenchyma through the periarterial space, where the interstitial fluid then transports the metabolic waste to the meningeal lymphatics and deep cervical lymph nodes. It has been suggested that lymphatic dysfunction represents a common pathway for the accumulation of pathological proteins, which leads to clinical symptoms in primary neurodegenerative diseases [5, 6]. Although tau protein is an intracellular pathology characterized in PSP, intracellular tau can be released into extracellular space and subsequently eliminated from the brain via the glymphatic system [5].

Positron emission tomography (PET) can capture pathological changes associated with PSP. Previous studies have shown that dopamine transporter (DAT) PET imaging can illustrate dopaminergic system damage in vivo and have demonstrated high sensitivity for differentiating patients with PSP and other parkinsonism [7, 8]. Recently, several studies have demonstrated that new tau PET tracers can enhance diagnostic accuracy and facilitate the tracking of tau pathology in vivo in PSP [9, 10]. Furthermore, the brain metabolic dysfunction in PSP occurs at a system level beyond tau pathology and dopaminergic deficits. ^18^F‐Fluorodeoxyglucose (^18^F‐FDG) PET imaging can evaluate the metabolic abnormalities and play a crucial role in the early differential diagnosis of neurodegenerative parkinsonism. The network abnormality is specifically designed to detect unique interregional correlations by covariance analysis techniques in brain glucose metabolism, termed the PSP‐related pattern (PSPRP). It can provide a deeper understanding of the underlying pathological mechanisms [11, 12]. Specially, the diffusion tensor image analysis along the perivascular space (DTI‐ALPS) index can serve as a marker for the function of the glymphatic system. Previous studies have provided supporting evidence of glymphatic system impairment by the decreased ALPS index in PSP and demonstrated a strong relationship between the decreased ALPS index and tau deposition [13, 14]. It is possible that the imbalance between tau pathology production and clearance, resulting in tau accumulation and consequent impairment of dopaminergic neurons, plays a crucial role in the pathogenesis of PSP. However, the role of the glymphatic system in dopaminergic impairment and abnormal glucose metabolism in PSP remains largely unknown, in addition to its relationship with tau deposition.

In this study, we explored the associations of glymphatic activity with tau deposition, DAT loss, abnormal glucose metabolism, and motor dysfunction, and examined the role of tau deposition, dopaminergic impairment, and glucose metabolism in vivo on the relationship between glymphatic activity and clinical severity.

## 2 Material and Methods

### 2.1 Participants

Thirty‐eight patients with PSP and 18 normal controls (NC) were enrolled from the Huashan Parkinsonian PET Imaging (HPPI) Database, established by the Department of Nuclear Medicine & PET Center, Huashan Hospital, Fudan University. All PSP patients were diagnosed according to the latest clinical diagnostic criteria set forth by the Movement Disorder Society (MDS) [2]. Each patient underwent a comprehensive review of their medical history and physical examination by specialists in movement disorders. The PSP rating scale (PSPrs) and Part III of the unified Parkinson's disease rating scale (UPDRS III) were utilized to assess the clinical severity [15, 16]. Participants with a history of structural brain abnormalities were excluded from the study. Due to the sensitivity of the ALPS index to the anterior commissure to posterior commissure (AC‐PC) line, images with an AC‐PC line exceeding the horizontal plane by more than 20° were also excluded. This study adhered to national and international regulations, including the 1964 Declaration of Helsinki and its subsequent amendments, as well as comparable ethical standards concerning human participants. The ethical permission for this study was granted by the institutional review board of Huashan Hospital (No. KY2019‐284), and all participants provided informed consent following a detailed explanation of the study.

### 2.2 Image Acquisition

After stopping taking anti‐parkinsonian medications for at least 12 h, all patients with PSP initially underwent PET and magnetic resonance imaging (MRI) data collection. For ^18^F‐Florzolotau PET, only 16 NC completed the data collection. For ^18^F‐FDG PET, only 11 NC completed the data collection. For ^18^F‐FPCIT PET, data collection was not completed for any NC.

^18^F‐Florzolotau and ^18^F‐FDG PET data were acquired on a Biograph mCT Flow PET/CT scanner (Siemens Healthcare GmbH, Erlangen, Germany). Initially, a 10‐s low‐dose CT scan was performed for attenuation correction. After the transmission scan, the emission scan was conducted 90 min after the injection of approximately 259 MBq ^18^F‐Florzolotau (over a duration of 20 min) or 60 min after the injection of approximately 185 MBq ^18^F‐FDG (over a duration of 10 min). PET images were reconstructed in three dimensions using the ordered subset expectation maximization (OSEM) algorithm.

^18^F‐FPCIT PET and DTI data were obtained on a uPMR 790 T PET/MR scanner (United Imaging, Shanghai, China). A 20‐min static PET scan started at 60 min postinjection of approximately 148 MBq. The T1‐weighted MR and DTI scans were conducted with the following parameters: T1: TR/TE, 7.19 ms/3.0 ms; flip angle, 10°; trans‐axial acquisition matrix, 256 × 230; in‐plane resolution, 1 × 1 mm; slice thickness, 1 mm; sagittal slice, 160. DTI: TR/TE, 8632 ms/84.9 ms; voxel size, 2.5 × 2.5 × 2.5 mm; 65 volumes (64 b = 1000 s/mm^2^ + 1 b = 0 s/mm^2^); flip angle, 90°.

### 2.3 DTI‐ALPS Index Analysis

Raw DTI images were initially converted to NIFTI format, followed by a comprehensive inspection for the bvec and bval. Subsequent preprocessing steps included susceptibility‐induced distortion correction, brain extraction, and simultaneous correction for eddy currents and subject motion. The DTI model was then fitted based on the corrected images, and all tensor images were registered to the MNI space. This processing pipeline was implemented using FMRIB's Diffusion Toolbox (FDT), in accordance with the guidelines provided by the FMRIB Software Library (https://fsl.fmrib.ox.ac.uk).

Using the JHU‐ICBM‐DTI‐81 white matter atlas, we identified two fiber tracts in the body of the lateral ventricle: the superior longitudinal fasciculus (SLF) and the superior corona radiata (SCR), namely the projection fibers and the associated fibers, respectively [17, 18]. The SLF (*y*‐axis) and SCR (*z*‐axis) are orthogonal to each other and aligned perpendicular to the *x*‐axis [18]. Previous studies have shown that medullary veins play an essential role in the clearance of the glymphatic system, which runs along the *x*‐axis in the plane of the lateral ventricle body (Dxxproj and Dxxassoc) [19]. Therefore, Dxxproj or Dxxassoc can approximately quantify the diffusivity in the perivascular space surrounding the medullary veins, reflecting glymphatic system activity. However, to account for the influence of intact white matter fiber tracts and variations due to different scanning parameters and equipment, we calculated the diffusivity along the *y* and *z* axes of the SLF and SCR (Dyyproj and Dzzassoc) to neutralize their effects [20, 21]. Finally, the ALPS index in the left/right hemisphere was calculated according to the following formula. The mean ALPS index was also calculated based on bilateral hemispheres. A higher ALPS index indicates more active movement of water molecules along the medullary veins and better glymphatic function, whereas a lower ALPS index may suggest impairment of glymphatic function.

$$\text{ALPS index}=\frac{\text{Mean}\left(D_{\text{xxproj}},D_{\text{xxassoc}}\right)}{\text{Mean}\left(D_{\text{yyproj}},D_{\text{zzassoc}}\right)}$$

### 2.4 PET Imaging Analysis

The original ^18^F‐Florzolotau, ^18^F‐FPCIT, and ^18^F‐FDG PET (tau‐PET, FPCIT‐PET, and FDG‐PET) images and T1‐weighted MRI images were checked first. Then, the PET images were registered to the corresponding T1‐weighted MRI images, followed by spatial normalization, aligning to MNI space. Finally, a Gaussian kernel (full‐width at half‐maximum: 6 mm) was applied to smooth the registration error. This processing pipeline was implemented using the specialized brain imaging analysis software package, SPM12 (https://www.fil.ion.ucl.ac.uk/). The processed tau‐PET images were analyzed by calculating the standardized uptake value ratio (SUVR) for the region of interest (ROI), using the cerebellar gray matter as the reference region [9]. The ROIs were composed of cortical and subcortical regions. The cortical regions included the frontal, temporal, parietal, and occipital lobes, while the subcortical regions included the caudate, putamen, external and internal globus pallidus, thalamus, midbrain, red nucleus, raphe nucleus, dentate nucleus, locus coeruleus, substantia nigra, and subthalamic nucleus [22]. The processed FPCIT‐PET images were calculated for semi‐quantification by using a previously defined volume of interest (VOI) atlas, which consisted of bilateral caudate, bilateral anterior putamen, bilateral posterior putamen, and occipital area [23]. The specific‐to‐nonspecific binding ratio (SNBR) was defined as follows: (mean radioactive counts of the striatal subregional VOI—mean radioactive counts of the occipital VOI)/mean radioactive counts of the occipital VOI, with the occipital uptake considered as nonspecific binding. The processed FDG‐PET images were analyzed by scaled subprofile modeling/principal component analysis (SSM/PCA) to identify abnormal metabolic brain network activity, referred to as PSPRP. The identification of PSPRP was described previously [24]. The subject expression scores for PSPRP were calculated based on this identification.

### 2.5 Statistics Analysis

Data normality was checked using the Kolmogorov–Smirnov test. A two‐sample t‐test was used to compare the demographic data, ALPS index, tau‐PET SUVR, and PSPRP expression scores between the PSP and NC groups. The chi‐square test was used for group comparisons of the categorical variable, gender. All correlation analyses were conducted using Pearson correlation with age and sex as covariates. To further explore the role of tau deposition, dopaminergic impairment, and glucose metabolism on the relationship between the ALPS index and clinical scale scores, six mediation pathways were examined. The significance of the mediation effects was tested using the bootstrap method, with 5000 resamples and a 95% confidence interval. The analysis was conducted using SPSS for Windows (v26.0; IBM, Armonk, NY, USA). Unless otherwise specified, all hypothesis tests were two‐tailed, with statistical significance defined as a *p* < 0.05.

## 3 Results

### 3.1 Demographics

The clinical and demographic information of PSP patients is presented in Table 1. PSP patients were significantly older than the NC group (*p* < 0.05). The clinical scores of PSP patients showed significant differences, with higher PSPrs and UPDRS III scores compared with the NC group. There was no significant difference between PSP patients and NC groups regarding gender (*p* > 0.05).

**TABLE 1:** The clinical and demographic information of patients with PSP.

	PSP	NC	*p*
Gender (M/F)	27/11	8/10	0.055
Age (years)	68.55 ± 6.92	60.83 ± 9.26	0.001
PSPrs scores	32.05 ± 14.16	0	< 0.001
UPDRS III scores	39.39 ± 16.43	0	< 0.001
ALPS index‐Left	1.03 ± 0.19	1.18 ± 0.23	0.011
ALPS index‐Right	0.94 ± 0.17	1.31 ± 0.17	< 0.001
ALPS index‐Bilateral	0.98 ± 0.16	1.16 ± 0.18	< 0.001
PSPRP	4.88 ± 2.10	1.98 ± 0.94	< 0.001

Abbreviations: ALPS, analysis along the perivascular space; H&Y, Hoehn and Yahr; NC, normal control; PSP, progressive supranuclear palsy; PSPRP, PSP‐related pattern; PSPrs, PSP rating scale; UPDRS III, Unified Parkinson's Disease Rating Scale III.

### 3.2 The DTI‐ALPS/PET Indexes in PSP

**FIGURE 1:**
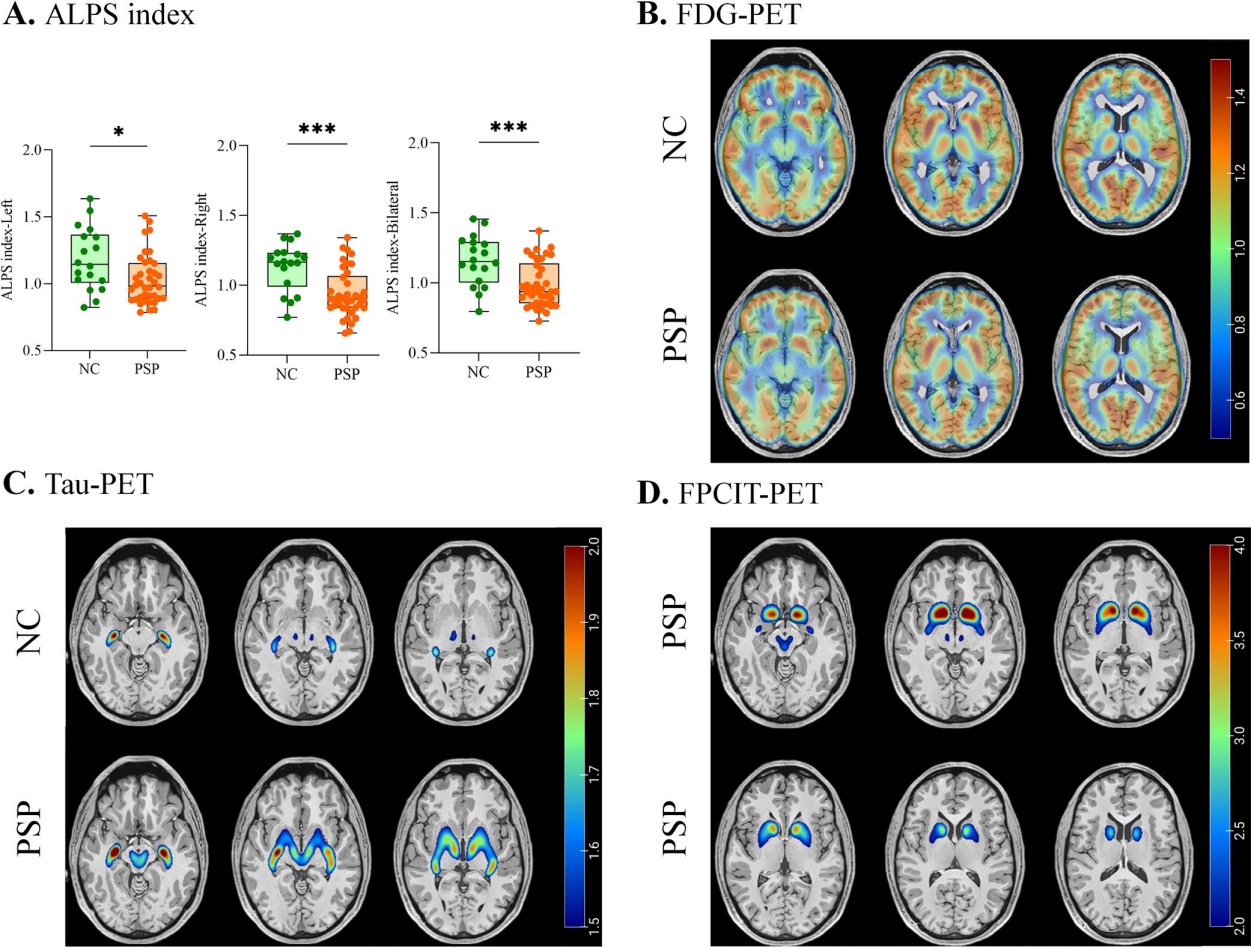
The ALPS index and PET profiles in PSP. (A) Comparisons of the ALPS index between the PSP and NC groups. (B) The averaged ^18^F‐FDG PET SUVR images in the PSP and NC groups. (C) The averaged ^18^F‐Florzolotau PET SUVR images in the PSP and NC groups. (D) The averaged ^18^F‐FPCIT PET SUVR images in PSP. NC, normal control; PSP, progressive supranuclear palsy; SUVR, standardized uptake value ratio.

The abnormal alterations in diffusivity may be caused by the damage to the glymphatic system, which could be reflected by the decreased ALPS index. As shown in Table 1 and Figure 1A, the ALPS index were significantly lower in the PSP patients compared to the NC group in the left hemisphere, right hemisphere, and bilateral brain averages, suggesting potential glymphatic dysfunction in PSP. Although no obvious differences in FDG‐PET images from PSP patients and the NC group were visually presented in Figure 1B, the PSPRP, which represents the global metabolic pattern, captured metabolic network abnormalities (Table 1, *p* < 0.001). Additionally, compared to the NC group, the tau‐PET SUVR of the PSP patients showed significant abnormal tau deposition in the cortical regions (frontal, parietal, and occipital lobe) (Table 2, *p* < 0.05) and subcortical regions (caudate, putamen, globus pallidus, thalamus, red nucleus, raphe nucleus, locus coeruleus, subthalamic nucleus, and midbrain) (Figure 1C and Table 2, *p* < 0.05). Since the NC did not undergo FPCIT‐PET imaging, we only displayed the averaged FPCIT‐PET SUVR images for the PSP patients (Figure 1D). It is still evident that the PSP patients exhibited significant dopamine transporter abnormalities in the basal ganglia, where normal individuals should show a homogeneous, intact bean sprout shape without abnormal reductions. Figure 1D showed clear dopamine transporter abnormalities, characterized by reductions in regions such as the caudate and putamen.

**TABLE 2:** The comparison of tau PET SUVR between PSP and NC.

	PSP	NC	*p*
*Cortical regions*			
Frontal	1.05 ± 0.19	0.94 ± 0.07	0.002
Parietal	1.07 ± 0.19	0.95 ± 0.07	0.006
Temporal	1.09 ± 0.17	1.04 ± 0.08	0.079
Occipital	1.11 ± 0.17	1.05 ± 0.07	0.042
*Subcortical regions*			
Caudate	1.09 ± 0.33	0.96 ± 0.13	0.044
Putamen	1.42 ± 0.26	1.15 ± 0.10	< 0.001
GPe	1.66 ± 0.27	1.25 ± 0.11	< 0.001
GPi	1.71 ± 0.26	1.31 ± 0.14	< 0.001
Thalamus	1.62 ± 0.28	1.41 ± 0.22	0.010
Midbrain	1.44 ± 0.16	1.25 ± 0.13	< 0.001
Red Nucleus	1.71 ± 0.35	1.43 ± 0.15	0.002
Raphe nucleus	1.65 ± 0.28	1.38 ± 0.14	< 0.001
Dentate nucleus	1.49 ± 0.22	1.40 ± 0.15	0.076
Locus coeruleus	1.51 ± 0.28	1.31 ± 1.43	0.001
Substantia nigra	1.44 ± 0.30	1.34 ± 0.17	0.119
Subthalamic nucleus	1.71 ± 0.35	1.41 ± 0.16	0.001

Abbreviations: GPe, external globus pallidus; GPi, internal globus pallidus; NC, normal control; PSP, progressive supranuclear palsy; SUVR, standardized uptake value ratio.

### 3.3 The Associations Between DTI/PET Indexes and Clinical Scores

**FIGURE 2:**
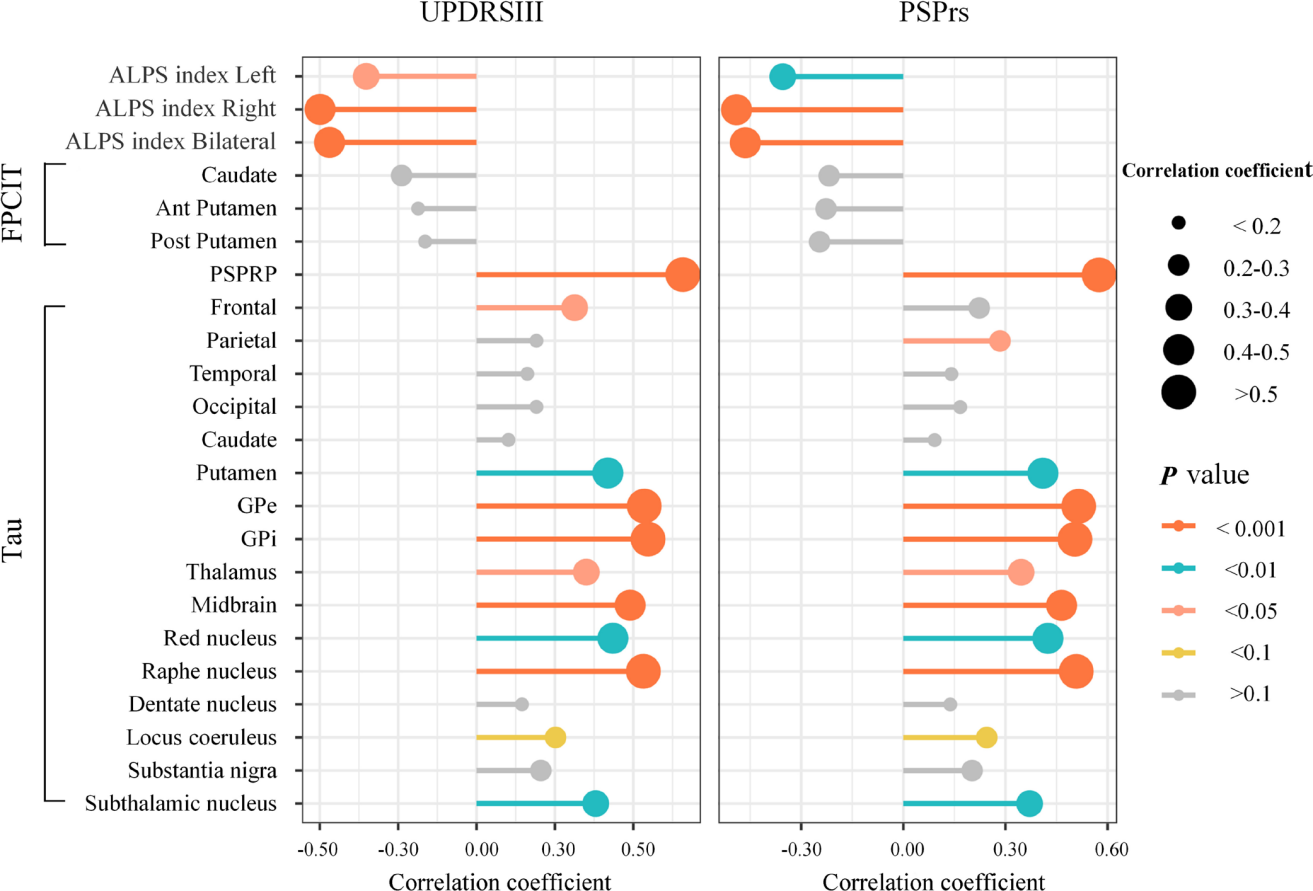
The correlations between the ALPS index, FPCIT SNBR, PSPRP, tau PET SUVR, and clinical severity. ALPS, analysis along the perivascular space; Ant, anterior; GPe, external globus pallidus; GPi, internal globus pallidus; Post, posterior; PSPRP, PSP‐related pattern; SNBR, specific to nonspecific binding ratio; SUVR, standardized uptake value ratio.

Figure 2 displayed the correlations between ALPS index, FPCIT‐PET SNBR, PSPRP expression scores, tau‐PET SUVR, and clinical scores, including UPDRS III and PSPrs. As presented above, the ALPS indexes were significantly negatively correlated with both UPDRS III and PSPrs in the left hemisphere, right hemisphere, and bilateral brain averages (*p* < 0.01). No significant correlations were found between FPCIT‐PET SNBR and clinical scores. PSPRP expression scores showed the strongest correlation with both UPDRS III and PSPrs (*r* = 0.658, *p* < 0.001; *r* = 0.575, *p* < 0.001, respectively). For tau‐PET, the SUVR in the frontal lobe was significantly positively correlated with UPDRS III scores (*r* = 0.313, *p* = 0.022), and the SUVR in the parietal lobe was significantly positively correlated with PSPrs (*r* = 0.284, *p* = 0.036). No significant correlations were found in other cortical regions. Various tau‐PET SUVRs in subcortical nuclei showed varying degrees of positive correlations with UPDRS III and PSPrs, including the putamen, external globus pallidus (GPe), internal globus pallidus (GPi), thalamus, midbrain, red nucleus, raphe nucleus, and subthalamic nucleus (all *p* < 0.05).

### 3.4 The Associations Between DTI‐ALPS and PET Indexes

**FIGURE 3:**
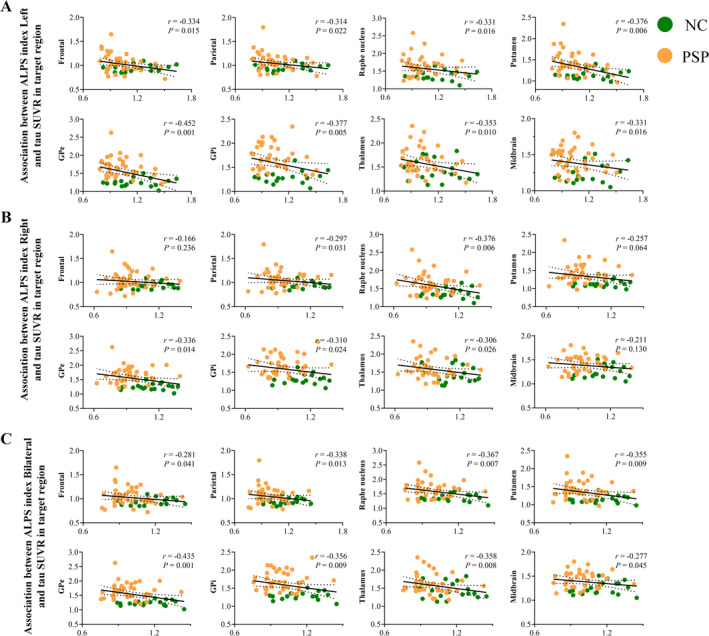
The correlations between the ALPS index and tau PET SUVR in brain regions.(A) ALPS index‐Left shows significantly negative correlations with tau‐PET SUVR in brain regions. (B) ALPS index‐Right shows significantly negative correlations with tau‐PET SUVR in brain regions. (C) ALPS index‐Bilateral shows significantly negative correlations with tau‐PET SUVR in brain regions. Note: SUVR, standardized uptake value ratio; GPe, external globus pallidus; GPi, internal globus pallidus.

As shown in Figure 3, there were varying degrees of significant negative correlations between ALPS indexes and tau‐PET SUVR in the frontal and parietal lobes. Among these, the strongest correlation was found between ALPS index‐Bilateral and tau‐PET SUVR in the parietal lobe (*r* = −0.338, *p* = 0.013). However, no significant correlation were found between ALPS indexes and tau‐PET SUVR in the occipital and temporal lobes (Table S1). Notably, significant negative correlations were observed between ALPS indexes and tau deposition in several subcortical regions including the putamen, GPe/GPi, thalamus, midbrain, red nucleus, raphe nucleus, and locus coeruleus (Figure 3 and Table S1, *p* < 0.05). Among these, the strongest correlation was found between ALPS index‐Left and tau‐PET SUVR in GPe (*r* = −0.452, *p* = 0.001). Additionally, the tau‐PET SUVR in the red nucleus and locus coeruleus was significantly correlated only with ALPS index in the left hemisphere. No significant correlations were found between tau‐PET SUVR in other subcortical nuclei and the ALPS indexes (Table S1).

In addition, we investigated the association between the ALPS indexes and FPCIT‐PET SNBR and PSPRP expression in PSP patients. ALPS indexes did not correlate with the DAT activities in any striatal subregion (Figure 3). However, it is noteworthy that we found a significant negative correlations between ALPS indexes and PSPRP expression (ALPS index‐Left: *r* = −0.347, *p* = 0.018; ALPS index‐Right: *r* = −0.446, *p* = 0.002; ALPS index‐Bilateral: *r* = −0.347, *p* = 0.018) (Figure 3).

### 3.5 Mediation Analysis

**FIGURE 4:**
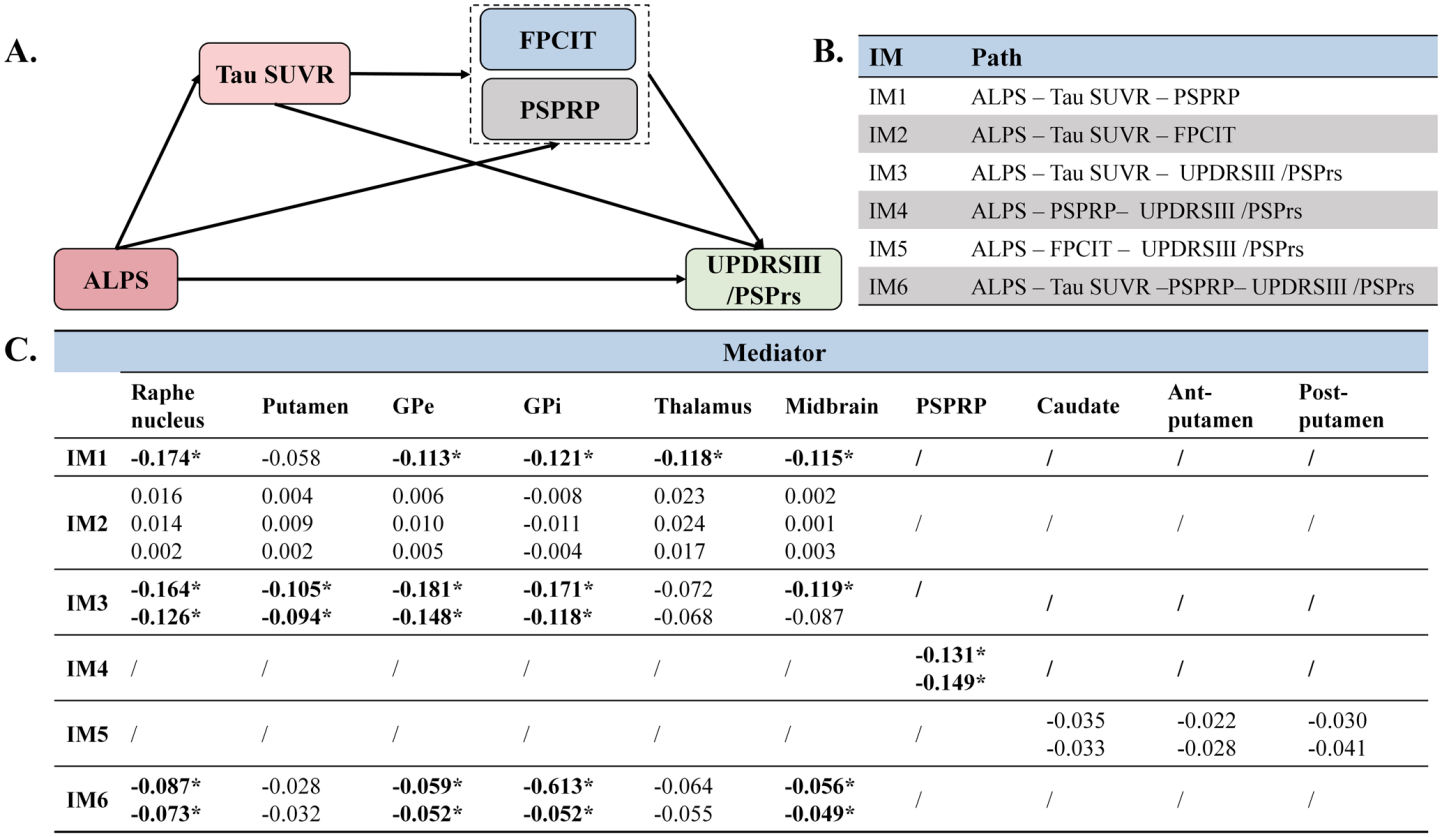
Mediation analysis showing tau deposition in the subcortical regions and PSPRP as significant mediators between the ALPS index‐Bilateral and clinical severity. (A) Illustrations of mediation analysis of PET indexes in the relationship between ALPS index and clinical severity (B) path lists (IM1‐IM6) in conducting mediation analysis (C) table of standardized coefficients on six pathways. For tau SUVR, the mediating role of cortical and subcortical tau burden on the relationship between ALPS index and other PET indexes or clinical severity was explored. For FPCIT, the mediating role of caudate, anterior putamen, and posterior putamen on the relationship between ALPS index and clinical severity was explored. Ant, anterior; GPe, external globus pallidus; GPi, internal globus pallidus; Post, posterior; PSPRP, PSP‐related pattern. *p < 0.05.

We conducted mediation analysis to further investigate the role of PSP‐associated hallmarks in the relationship between glymphatic system activity and clinical severity. As illustrated in Figure 4A‐B, there are six pathways: IM1: ALPS–Tau SUVR–PSPRP, IM2: ALPS–Tau SUVR–FPCIT, IM3: ALPS–Tau SUVR–UPDRS III/PSPrs, IM4: ALPS–PSPRP–UPDRS III/PSPrs, IM5: ALPS–FPCIT–UPDRS III/PSPrs, and IM6: ALPS–Tau SUVR–PSPRP–UPDRS III/PSPrs. IM1 and IM2 were constructed to explore the potential associations between ALPS and PET indexes. These results in Figure 4C indicated that the ALPS index may influence global glucose metabolism through subcortical tau deposition, with standardized coefficients ranging from −0.113 to −0.174. However, no significant association was found between dopaminergic impairment and tau deposition or the ALPS index. Subsequently, IM3, IM4, and IM5 were used to investigate the effects of tau‐PET SUVR, PSPRP expression, and FPCIT‐PET SNBR on the relationship between the ALPS index and clinical scores. Similarly, FPCIT‐PET SNBR was not involved in this pathway, whereas PSPRP expression and subcortical tau‐PET SUVR demonstrated significant mediating effects between the ALPS index and clinical scores. Finally, the possibility of a cascading mediation effect pathway (IM6) was explored, revealing that although the standardized coefficients were smaller than those in IM3 and IM4, they remained significant. These findings in Figure 4 and Table S2–S3 indicated that subcortical tau deposition and abnormal glucose metabolism secondary to glymphatic dysfunction may contribute to motor dysfunction.

## 4 Discussion

In this study, we found that PSP patients were characterized by a significantly decreased ALPS index, tau accumulation, dopaminergic dysfunction, and abnormal glucose metabolism in the brain. Moreover, our findings revealed that the ALPS index was correlated with tau accumulation and glucose metabolism in PSP patients but showed no association with dopaminergic dysfunction. Notably, we demonstrated that tau deposition and glucose metabolism, rather than the nigrostriatal system, were significant mediators between glymphatic dysfunction and clinical severity. To the best of our knowledge, this is the first study to evaluate the relationships between the deposition of tau protein and dopaminergic impairment and abnormal glucose metabolism, and glymphatic system activity estimated by the DTI‐ALPS technique.

The glymphatic system is a clearance pathway for soluble proteins, and its impairment contributes to tau aggregation and neurodegeneration. The ALPS index is an indicator related to the glymphatic system, which is evaluated by the whole‐brain glymphatic activity. Several studies showed the reproducibility of DTI‐ALPS and its relationship with glymphatic clearance ability, as calculated by intrathecal injections of gadolinium‐based contrast agents [25, 26]. In this study, we confirmed that the ALPS index is obviously decreased in PSP patients. This finding is consistent with previous reports [13, 14], and suggests potential glymphatic dysfunction in PSP patients. Additionally, we found significant abnormal deposition of tau in the cortical regions, as well as subcortical regions, and abnormal metabolic brain network activity in PSP patients compared with NC. It is possible that the abnormal metabolic brain network may be attributed, in part, to more widespread tau pathology. Furthermore, the pattern of DAT imaging revealed abnormal dopaminergic function in PSP patients. It is in tune with pathologic reports, showing that PSP patients have more tau pathology in the deep nuclei of subcortical regions [27]. Thus, tau accumulation in the subcortical nucleus, particularly in the substantia nigra and striatum, may cause greater nigrostriatal dysfunction and the clinical symptoms of parkinsonism in PSP. Although the underlying pathomechanisms for the PSP‐associated hallmarks remain unclear, our study suggests the coexisting of these alterations in PSP patients.

Previous studies suggested the association between disease incidence and age/sex in PSP. It may result in bias in correlation analysis or mediation analysis. Considering that, we assessed the correlation and mediation effect between ALPS index/PET indexes and clinical scores with age and sex as covariates. A study has demonstrated the association between tau deposition and glymphatic function using in vivo imaging methods [13]. In ROI analysis, our study revealed that the decreased ALPS indexes were related to increased tau aggregation. The evidences provided by these results suggests that glymphatic dysfunction is associated with tau pathology. It could be thought that glymphatic system activity might contribute to the tau deposition observed in PSP, though further evidence is required. Yet, there was no significant relationship between the ALPS index and FPCIT‐PET SNBR in the whole striatum in our study. FPCIT‐PET directly reflects the DAT expression and dopaminergic function. The lack of a significant correlation between ALPS index and dopaminergic dysfunction may partly result from the course of disease progression. In our study, PSP patients showed diffuse and symmetric DAT loss across the entire striatum. These results accord well with the incipient floor effect observed in striatal DAT loss. As the ALPS index continued to decline, the trend of decreasing DAT binding slowed, and the correlation correspondingly diminished. The overall weak correlations between the two measures may suggest that they represent different pathophysiological mechanisms of the disease. Furthermore, PSPRP expression was negatively correlated with ALPS index. Evidence suggests that increased PSPRP expression is associated with more severe disease [28]. It provides a deeper comprehension of the pathophysiological mechanisms by brain metabolic network [12]. As we observed that ALPS index may influence clinical severity through network changes in PSPRP. And, other studies have indicated that disrupting glymphatic pathways promotes tau accumulation [29, 30]. Considering these findings, we believe that the ALPS index is sensitive to PSP compared to other biomarkers that may reflect later‐occurring pathologies. Glymphatic function reflected by the ALPS index may play a more significant role in the early detection and have translational potential to therapeutically relieve neurological disorders [31].

Tau deposition has been hypothesized to mediate glymphatic activity and clinical severity in PSP [13]. However, the contributions of dopaminergic impairment and abnormal glucose metabolism on the relationships between the ALPS indexes and clinical scores have not been previously reported. We conducted a mediation analyses to delve deeper into the intricate relationship between the glymphatic system, PSP‐associated hallmarks, and clinical severity. The results revealed that subcortical tau deposition and abnormal metabolic brain network activity play a mediating role between the glymphatic system and clinical severity. It suggests that the impairment of the glymphatic system may be involved in tau toxicity and global metabolic changes, resulting in motor symptoms. However, tau deposition in any cortical region was not associated with the ALPS index in this study. It is noteworthy that tau accumulation in the subcortical region, rather than in the cortical region, should be responsible for motor dysfunction. A recent study suggested that remote cortical dysfunction may be caused by subcortical tau pathology, which could lead to clinical manifestation [32]. In contrast to our results, another study revealed that glymphatic activity and clinical severity are influenced by the presence of tau burden in cortical regions rather than in subcortical regions [13]. The unbalanced distribution of PSP phenotypes in these studies may lead to different results. In our study, most PSP phenotypes are PSP‐RS, which has more subcortical tau deposition than other variant PSP syndromes [22, 33]. Further studies with larger sample sizes and various phenotypes are needed to further explore the relationships.

This study has several limitations. First, the diagnoses for all patients enrolled in this study were determined based on clinical diagnostic criteria rather than pathologic studies. We selected patients with follow‐up to avoid misdiagnosis. Second, the study was conducted with a relatively small sample size through a cross‐sectional analysis, which restricts the generalizability of our findings. Nevertheless, we opted for rigorous inclusion and exclusion criteria to ensure uniformity in the clinical features of the patients who were enrolled. Finally, this study was conducted at a single center, and the potential bias should be acknowledged. It is essential to validate these findings in future studies involving larger samples across multiple centers and with longitudinal follow‐up. In addition, the results of mediator effect were based on analysis came from statistical analysis, not the real physiological mechanism, and need to be interpreted carefully.

In conclusion, our results revealed more severe glymphatic dysfunction, tau deposition, DAT loss, and abnormal metabolic brain network activity in PSP. The ALPS index was negatively associated with tau deposition and PSPRP expression. Subcortical tau deposition and PSPRP expression mediate glymphatic system activity and clinical severity, suggesting that tau deposition secondary to lower glymphatic availability may accelerate abnormal metabolic brain network activity, leading to further motor dysfunction. These collective findings highlight that PSP is both complex and multifaceted, which demands a deeper exploration of the temporal relationships among the various pathophysiological factors involved in this disease. Our results provide more neuroimaging evidence and support the growing understanding of the pathogenesis of PSP.

## Author Contributions

Fangyang Jiao: Conceptualization, investigation, methodology, resources, visualization, and original draft. Qingmin Wang: Conceptualization, methodology, resources, and review and editing. Jiayi Zhong: Conceptualization, methodology, visualization, and review and editing. Huamei Lin: Resources, Review and editing. Jiaying Lu: Resources, Review and editing. Luyao Wang: Methodology, Review and editing. Min Wang: Methodology, Review and editing. Fengtao Liu: Resources, supervision, and review and editing. Jiehui Jiang: Conceptualization, funding acquisition, resources, supervision, visualization, and review and editing. Chuantao Zuo: Conceptualization, funding acquisition, resources, supervision, visualization, and review and editing.

## Conflicts of Interest

The authors declare no conflicts of interest.

## Supporting information


Table S1

## Data Availability

The data that support the findings of this study are available from the corresponding author upon reasonable request.
