# Supplementary material for: Relationships Between Glymphatic System Activity and Tau Burden, Dopaminergic Impairment, Abnormal Glucose Metabolism in Progressive Supranuclear Palsy
Source: CNS Neurosci Ther. 2025 Feb 18;31(2):e70284. doi: 10.1111/cns.70284 (PMC11833299; doi:10.1111/cns.70284)
Supplement: Supplementary file 1 — Table S1 [file CNS-31-e70284-s001.docx]

# Supplementary information

Table S1. The correlations between DTI-ALPS index and tau PET SUVR.

|  | ALPS index Left | | ALPS index Right | | ALPS index Bilateral | |
| --- | --- | --- | --- | --- | --- | --- |
|  | *r* | *P* | *r* | *P* | *r* | *P* |
| Frontal | *-0.334* | *0.015* | *-0.166* | *0.236* | *-0.281* | *0.041* |
| Parietal | *-0.314* | *0.022* | *-0.297* | *0.031* | *-0.338* | *0.013* |
| Temporal | *-0.072* | *0.607* | *0.095* | *0.497* | *0.015* | *0.914* |
| Occipital | *-0.147* | *0.294* | *-0.079* | *0.576* | *-0.121* | *0.389* |
| Caudate | *-0.185* | *0.184* | *-0.001* | *0.993* | *-0.089* | *0.527* |
| Putamen | *-0.376* | *0.006* | *-0.257* | *0.064* | *-0.355* | *0.009* |
| GPe | *-0.452* | *0.001* | *-0.336* | *0.001* | *-0.435* | *0.001* |
| GPi | *-0.377* | *0.005* | *-0.310* | *0.024* | *-0.356* | *0.009* |
| Thalamus | *-0.353* | *0.010* | *-0.306* | *0.026* | *-0.358* | *0.008* |
| Midbrain | *-0.331* | *0.016* | *-0.221* | *0.130* | *-0.277* | *0.045* |
| Red Nucleus | *-0.304* | *0.027* | *-0.181* | *0.190* | *-0.231* | *0.092* |
| Raphe nucleus | *-0.331* | *0.016* | *-0.376* | *0.006* | *-0.367* | *0.007* |
| Dentate nucleus | *-0.251* | *0.070* | *-0.109* | *0.436* | *-0.199* | *0.154* |
| Locus_coeruleus | *-0.317* | *0.021* | *-0.140* | *0.317* | *-0.258* | *0.063* |
| Substantia nigra | *-0.001* | *0.995* | *0.099* | *0.481* | *0.077* | *0.586* |
| Subthalamic nucleus | *-0.197* | *0.157* | *-0.118* | *0.402* | *-0.089* | *0.527* |

Note: GPe, external globus pallidus; GPi, internal globus pallidus; ALPS, analysis along the perivascular space; SUVR, standardized uptake value ratio.

Table S2. Mediation analysis showing tau deposition in the subcortical regions and PSPRP as significant mediators between ALPS index-Left and clinical characteristics.

|  | Raphe nucleus | Putamen | GPe | GPi | Thalamus | Midbrain | PSPRP | Caudate | Ant-putamen | Post-putamen |
| --- | --- | --- | --- | --- | --- | --- | --- | --- | --- | --- |
| **IM1** | **-0.118*** | -0.064 | **-0.108*** | -0.112 | -0.104 | -0.106 | **/** | **/** | **/** | **/** |
| **IM2** | 0.011 | 0.005 | 0.018 | 0.005 | 0.024 | -0.015 | **/** | **/** | **/** | **/** |
|  | 0.009 | 0.017 | 0.028 | 0.007 | 0.024 | -0.011 |  |  |  |  |
|  | 0.005 | 0.005 | 0.018 | 0.003 | 0.017 | -0.020 |  |  |  |  |
| **IM3** | **-0.150*** | **-0.123*** | **-0.200*** | **-0.185*** | -0.084 | **-0.129*** | **/** | **/** | **/** | **/** |
|  | **-0.117*** | **-0.113*** | **-0.167*** | **-0.135*** | -0.079 | **-0.098*** |  |  |  |  |
| **IM4** | **/** | **/** | **/** | **/** | **/** | **/** | -0.116 | **/** | **/** | **/** |
|  |  |  |  |  |  |  | -0.103 |  |  |  |
| **IM5** | **/** | **/** | **/** | **/** | **/** | **/** | **/** | -0.055 | -0.034 | -0.015 |
|  |  |  |  |  |  |  |  | -0.042 | -0.045 | -0.022 |
| **IM6** | **-0.059*** | -0.037 | **-0.060*** | **-0.059*** | -0.057 | -0.055 | **/** | **/** | **/** | **/** |
|  | **-0.051*** | -0.034 | **-0.052*** | **-0.051*** | -0.050 | -0.048 |  |  |  |  |

Note: PSPRP, PSP-related pattern; GPe, external globus pallidus; GPi, internal globus pallidus; Ant, anterior; Post, posterior. * *P* < 0.05.

Table S3. Mediation analysis showing tau deposition in the subcortical regions and PSPRP as significant mediators between ALPS index-Right and clinical characteristics.

|  | Raphe nucleus | Putamen | GPe | GPi | Thalamus | Midbrain | PSPRP | Caudate | Ant-putamen | Post-putamen |
| --- | --- | --- | --- | --- | --- | --- | --- | --- | --- | --- |
| **IM1** | **-0.192*** | -0.045 | -0.096 | -0.104 | -0.107 | -0.098 | **/** | **/** | **/** | **/** |
| **IM2** | 0.019 | -0.003 | -0.012 | -0.024 | 0.016 | 0.013 | **/** | **/** | **/** | **/** |
|  | 0.016 | -0.006 | -0.016 | -0.028 | 0.017 | 0.010 |  |  |  |  |
|  | 0.029 | 0.004 | -0.007 | -0.004 | 0.010 | 0.025 |  |  |  |  |
| **IM3** | **-0.158*** | **-0.086*** | **-0.152*** | **-0.142*** | -0.064 | **-0.099*** | **/** | **/** | **/** | **/** |
|  | **-0.121*** | -0.073 | **-0.119*** | -0.091 | -0.059 | -0.067 |  |  |  |  |
| **IM4** | **/** | **/** | **/** | **/** | **/** | **/** | **-0.146*** | **/** | **/** | **/** |
|  |  |  |  |  |  |  | **-0.125*** |  |  |  |
| **IM5** | **/** | **/** | **/** | **/** | **/** | **/** | **/** | -0.020 | -0.007 | 0.039 |
|  |  |  |  |  |  |  |  | -0.018 | -0.008 | 0.054 |
| **IM6** | **-0.097*** | -0.024 | -0.048 | -0.051 | -0.056 | -0.045 | **/** | **/** | **/** | **/** |
|  | **-0.079*** | -0.021 | -0.041 | -0.042 | -0.047 | -0.038 |  |  |  |  |

Note: PSPRP, PSP-related pattern; GPe, external globus pallidus; GPi, internal globus pallidus; Ant, anterior; Post, posterior. * *P* < 0.05.
